# A hierarchical approach employing metabolic and gene expression profiles to identify the pathways that confer cytotoxicity in HepG2 cells

**DOI:** 10.1186/1752-0509-1-21

**Published:** 2007-05-11

**Authors:** Zheng Li, Shireesh Srivastava, Xuerui Yang, Sheenu Mittal, Paul Norton, James Resau, Brian Haab, Christina Chan

**Affiliations:** 1 Department of Chemical Engineering and Material Science, Michigan State University, East Lansing, MI 48824, USA; 2 Department of Biochemistry and Molecular Biology, Michigan State University, East Lansing, MI 48824, USA; 3 Department of Computer Science and Engineering, Michigan State University, East Lansing, MI 48824, USA; 4 Van Andel Institute, Grand Rapids, MI 49503, USA; 5 Department of Biochemistry and Molecular Biology, Michigan State University, USA

## Abstract

**Background:**

Free fatty acids (FFA) and tumor necrosis factor alpha (TNF-α) have been implicated in the pathogenesis of many obesity-related metabolic disorders. When human hepatoblastoma cells (HepG2) were exposed to different types of FFA and TNF-α, saturated fatty acid was found to be cytotoxic and its toxicity was exacerbated by TNF-α. In order to identify the processes associated with the toxicity of saturated FFA and TNF-α, the metabolic and gene expression profiles were measured to characterize the cellular states. A computational model was developed to integrate these disparate data to reveal the underlying pathways and mechanisms involved in saturated fatty acid toxicity.

**Results:**

A hierarchical framework consisting of three stages was developed to identify the processes and genes that regulate the toxicity. First, discriminant analysis identified that fatty acid oxidation and intracellular triglyceride accumulation were the most relevant in differentiating the cytotoxic phenotype. Second, gene set enrichment analysis (GSEA) was applied to the cDNA microarray data to identify the transcriptionally altered pathways and processes. Finally, the genes and gene sets that regulate the metabolic responses identified in step 1 were identified by integrating the expression of the enriched gene sets and the metabolic profiles with a multi-block partial least squares (MBPLS) regression model.

**Conclusion:**

The hierarchical approach suggested potential mechanisms involved in mediating the cytotoxic and cytoprotective pathways, as well as identified novel targets, such as NADH dehydrogenases, aldehyde dehydrogenases 1A1 (ALDH1A1) and endothelial membrane protein 3 (EMP3) as modulator of the toxic phenotypes. These predictions, as well as, some specific targets that were suggested by the analysis were experimentally validated.

## Background

Elevated levels of free fatty acids (FFAs) have been implicated in the pathogenesis of many obesity-related metabolic disorders [1-4], such as fatty liver disease and steatohepatitis. Dietary fatty acids produce a variety of metabolic and genetic effects on liver cells. Fatty acids compete with glucose for oxidation at the TCA cycle [5]. Fatty acids also cause changes in the enzyme make-up of the cells by regulating the transcription of enzymes of metabolism. FFAs exert their transcriptional effects by activating transcription factors (TFs) such as sterol receptor element binding protein (SREBP), peroxisome proliferator activated receptors (PPARs) and hepatic nuclear factors (HNFs) [6]. PPARs regulate the expression of proteins involved in fatty acid oxidation, SREBP regulates phospholipid and cholesterol synthesis and HNF affects both lipid and carbohydrate metabolism.

Tumor necrosis factor alpha (TNF-α) is another factor that has been shown to affect the function of hepatocytes in numerous ways. It has been associated with the development of hepatic insulin resistance and hepatocyte cell death [7-10]. TNF-α also activates transcription factors such as nuclear factor kappa B (NFκB) and c-Jun [11,12]. These transcription factors alter the expression of genes involved in cellular metabolism, cell proliferation and cell death [11,12]. Hepatocytes are known to be resistant to the cytotoxic action of TNF-α due to prompt upregulation of cytoprotective genes mediated by the activation of NF-κB in response to TNF-α [13]. Therefore, the cytotoxic effect of TNF-α requires a secondary insult, e.g., transcriptional inhibition [14] or glutathione depletion [15].

*In vivo*, under conditions of obesity, hepatocytes are simultaneously exposed to elevated FFAs and TNF-α. The importance of these factors in the pathogenesis of many diseases motivated this study of the physiological, metabolic and genetic effects of the simultaneous exposures to different types of FFAs and TNF-α. Among the many responses to FFA and TNF-α, the mechanism of cell death in response to simultaneous exposure to these factors is not well characterized. Hepatocyte cell death is suggested to play an important role in the development of various hepatic disorders, e.g. in non-alcoholic steatohepatitis (NASH). Previous studies on the toxic effects of different types of FFAs on liver cells have identified that saturated FFAs are much more toxic than unsaturated FFAs [16-19]. These studies have suggested that ER-ROS (reactive oxygen species) stress [17], mitochondrial alterations [18] and lysosomal permeabilization [19] are the major mechanisms in the toxicity of saturated FFAs. Studies on the toxicity of saturated FFAs to other cells have suggested that increased ROS production and ceramide synthesis [20,21] are the major mechanisms of palmitate-toxicity in those cells. Similarly, increased ceramide and ROS generation have been suggested to play important roles in the toxicity of TNF-α to hepatocytes [10,22]. However, there has not been any study on the cytotoxic effects of simultaneous exposure to FFAs and TNF-α.

**Figure 1:**
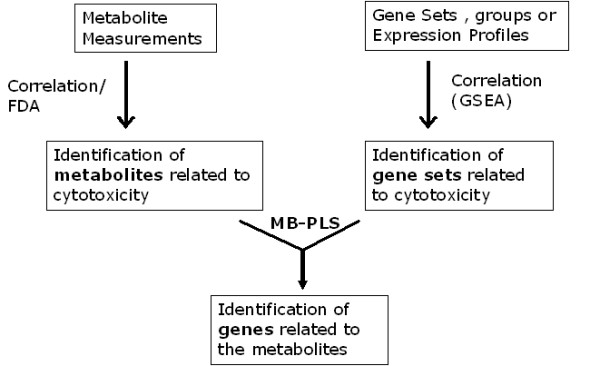
An overview of the hierarchical approach. First, important metabolic changes relevant to a phenotype were identified with discriminant analysis. Second, to identify the transcriptionally altered pathways, gene set enrichment analysis (GSEA) was applied to the cDNA microarray data. Finally, the expression of the enriched gene sets and the metabolic profiles were integrated with a multi-block partial least squares analysis (MBPLS) regression model to identify the genes and gene sets that regulate the metabolic pathways identified in step 1.

Human hepatoblastoma cells (HepG2) were treated simultaneously with different types of FFAs (saturated, monounsaturated and polyunsaturated) and levels of TNF-α and the cytotoxic, metabolic and genetic responses were studied. Exposing the cells to saturated fatty acid (palmitate) was cytotoxic and the exposure to TNF-α increased this toxicity, whereas the unsaturated FFAs induced increased triglyceride (TG) accumulation and were not cytotoxic. TNF-α alone was not toxic, either alone or in combination with the unsaturated FFAs. Our objective was to develop an approach to elucidate the underlying pathways that confer the cytotoxicity. The hierarchical framework developed to integrate the metabolic and genetic information to identify the genes and biological processes regulating a phenotypic response is shown in Figure 1. The framework consisted of three stages. First, the metabolic changes associated with the cytotoxic phenotype were identified with Fisher's Discriminant Analysis (FDA) [23]. Ketone body (e.g., beta-hydroxybutyrate (BOH) and acetoacetate (AcAc)) release and TG accumulation were identified to be the most important in separating the phenotypes, suggesting their involvement in inducing the cytotoxicity. To identify the signaling and gene regulatory pathways involved in regulating the toxicity, the genomic responses were measured using cDNA microarrays and analyzed with GSEA [24]. GSEA has been applied successfully to identify the signaling, transcriptional regulatory and metabolic pathways involved in the development of type 2 diabetes [25]. GSEA indicated the involvement of mitochondria-related, oxidative stress-related and FFA metabolism pathways in inducing the cytotoxic phenotype. Finally, the gene expression and metabolic profiles were integrated with multi-block partial least squares (MBPLS) regression analysis [26] to identify the genes most relevant to the metabolic changes which correlated most with the cytotoxicity. Some of the identified genes were experimentally perturbed to validate their predicted roles in regulating the cytotoxicity. This analysis identified that NADH dehydrogenases, aldehyde dehydrogenase 1A1 (ALDH1A1) and endothelial membrane protein 3 (EMP3) played important roles in the toxicity of FFAs.

## Results

### 1. Metabolic changes relevant to cytotoxicity

**Figure 2:**
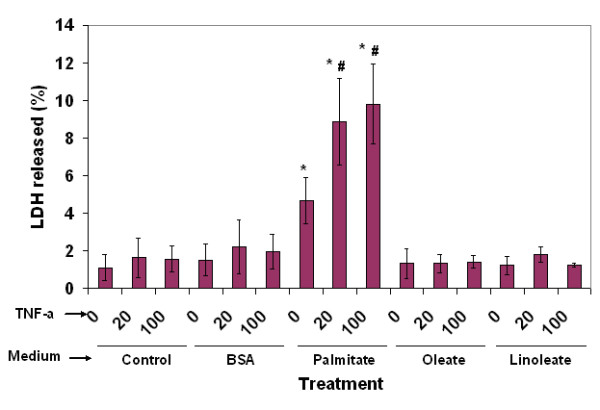
LDH release measurement of cytotoxicity of palmitate. Confluent HepG2 cells were treated with saturated (palmitate), monounsaturated (oleate) and polyunsaturated (linoleate) free fatty acid at 0.7 mM in presence or absence of TNF-α (0, 20 and 100 ng/ml) for 24 h. Cytotoxicity was measured as % LDH released. Data presented as average +/- s.d. of n = 9 from three independent experiments. *, significantly greater than control; #, significant TNF-α interaction effect (significantly greater than palmitate culture in absence of TNF-α. For both * and #, p < 0.01.

**Figure 3:**
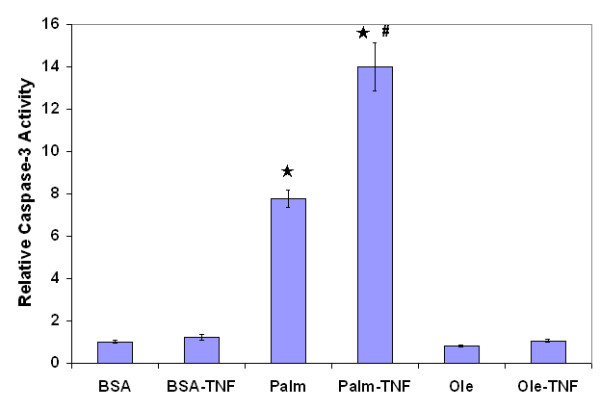
Caspase-3 activation measurement. Cells were treated for 24 h with different FFA in the presence or absence of 100 ng/ml TNF-α. At the end of incubation, cells were harvested and caspase-3 activity was measured fluorimetrically. Data presented as average +/- s.d. of n = 9 from three independent experiments. *, significantly greater than control; #, significant TNF-α interaction effect (significantly greater than palmitate culture in absence of TNF-α). For both * and #, p < 0.01.

**Figure 4:**
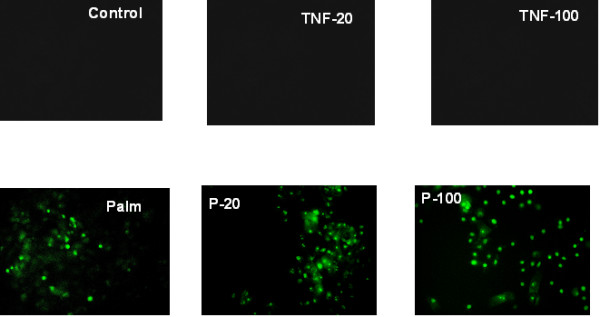
TUNEL assay measurement. Cells were treated as in Figure 2 followed by fluorimetric TUNEL assay using a commercially available kit.

**Figure 5:**
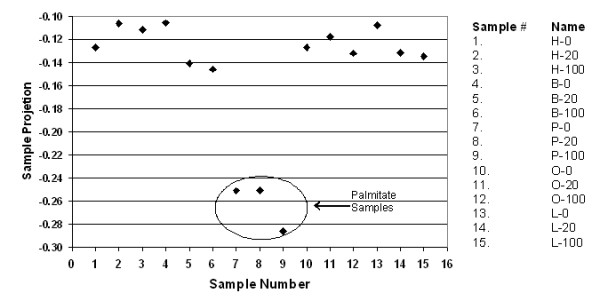
Fisher's discriminant analysis of metabolites. First FDA projection (T1) score plot of the samples.

**Figure 6:**
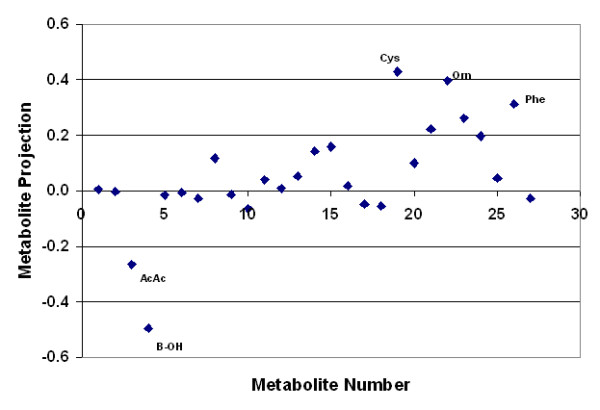
Fisher's discriminant analysis of metabolites. Loading plot of the coefficients of metabolites in T1.

**Figure 7:**
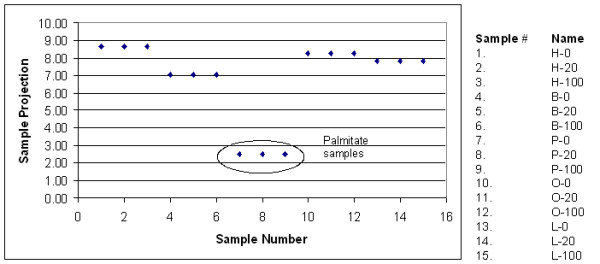
Fisher's discriminant analysis of metabolites. Second FDA projection (T2) score plot of the samples.

**Figure 8:**
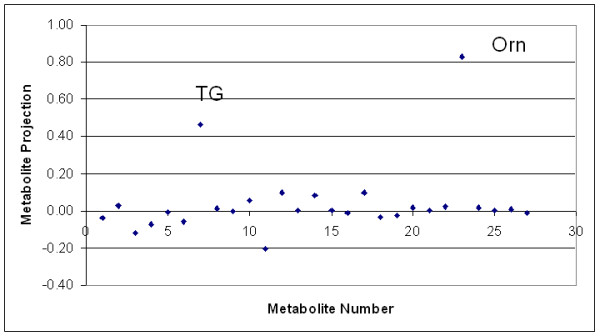
Fisher's discriminant analysis of metabolites. Loading plot of the coefficients of metabolites in T2.

As shown in Figure 2, the mono- and poly- unsaturated fatty acids, TNF-α and their combinations were found to be non-toxic. However, the saturated fatty acid palmitate was toxic to the cells and TNF-α increased this toxicity. Because the release of LDH is a late event in the process of cell death, we also measured the activation of caspase-3. Exposure to palmitate caused a significant elevation in the caspase-3 activity (Figure 3). Exposure to TNF-α significantly increased the caspase-3 activation only in the palmitate-treated cells, but not in the cells treated with control medium or oleate. These results indicated significant interactive effects of palmitate and TNF-α on the process of cell death. Finally, we also found that cells exposed to palmitate were also TUNEL positive (Figure 4), further corroborating with the evidence of initiation of apoptosis in these cells. Because palmitate was found to be the primary factor in the toxicity and the effects of TNF-α were only secondary to that of palmitate, we further investigated the mechanisms of toxicity of this FFA. To evaluate the metabolic changes responsible for mediating the toxicity, the rates of uptake/release of 27 metabolites, of glucose, fatty acid and amino acid metabolism, were measured and shown in additional data file 1. FDA was applied to identify the metabolic changes responsible for separating the phenotypic response (cytotoxic versus nontoxic as defined by the level of lactate dehydrogenase (LDH) release). As shown in Figure 5, the palmitate-treated samples were separated from the rest in a space defined by the first FDA projection (T1) with the palmitate samples taking on negative values while the rest of the samples taking on positive values on the Y axis. Furthermore, as shown in Figure 6 the production of BOH and acetoacetate, with the most negative coefficients, separated the palmitate samples in the direction of toxicity, while the uptake of cysteine, ornithine and phenylalanine, with the most positive coefficients, separated the samples away from the toxicity. In addition, the palmitate-treated samples were also separated from the rest of the sample in the space defined by the second FDA projection (T2) with the palmitate samples having smaller values than the rest of the samples as shown in Figure 7. Correspondingly, we found that TG and ornithine with the most positive coefficients in this projection separated the palmitate samples away from the toxicity as shown in Figure 8. Acetoacetate and BOH are the products of fatty acid oxidation. Increased fatty acid oxidation is known to increase oxidative stress and cell death [27,28]. TG accumulation has been found to protect cells from palmitate induced lipoxicity [29]. Thus, FDA analysis confirmed that fatty acid oxidation is positively associated with the palmitate-induced cytotoxicity, while accumulation of TG is negatively associated to cytotoxicity.

### 2. Functional pathway analysis with GSEA

37 gene sets involved in a variety of cellular processes and organelles which are known to play important roles in the changes caused by FFA and TNF-α were evaluated to identify which of these processes were associated with palmitate-induced cytotoxicity. The gene sets evaluated included those of fatty acid metabolism, cell death, TNF-α signaling, etc., (see Table 1 for a full list). 14 of the 37 gene sets were significantly enriched with nominal p values less than 0.05, and shown in Table 2. The enriched pathways included electron transport chain (ETC), fatty acid metabolism, glycolysis, oxidative phosphorylation, ROS, the pentose phosphate pathway (PPP), cell death, fatty acid beta-oxidation, TCA cycle, fructose metabolism, glutathione, and the ERK 1, ERK 2, MAP kinase pathways. This suggested that the toxicity may be associated with changes in oxidative stress related pathways such as ROS and glutathione, as well as energy generating processes of glycolysis, oxidative phosphorylation and ETC. Importantly, the gene set of sphingolipid (ceramide) metabolism was not selected, indicating that ceramide metabolism may not play an important role in the observed toxicity. Notably, most of the enriched gene sets belong to the mitochondria, for example fatty acid beta-oxidation, electron transport chain and oxidative phosphorylation, depicting a central role of mitochondria in the cytotoxicity. The gene set of mitochondria, which included 229 genes, was also significantly altered in the palmitate-treated cells, with a nominal p value of 0.00189.

### 3. Integrating the metabolic and the gene expression profiles to identify the pathways relevant to the cytotoxicity

To identify the genes that regulate the metabolic functions most closely associated with cytotoxicity, MBPLS models were developed to predict the metabolic processes based upon the expression data of the gene sets identified by GSEA. Ketogenesis and TG accumulation were identified to be positively and negatively related to LDH release in step 2, respectively. Therefore, two MBPLS models were developed to model BOH and TG, respectively. The MBPLS models contained 14 blocks each, corresponding to the 14 enriched gene sets identified by the GSEA. The importance of individual genes within these functional groups was identified by evaluating the regression coefficients of the genes. In particular, the genes with high positive regression coefficients to ketogenesis and TG accumulation are discussed below as the predicted roles of these genes were evaluated using inhibitors or RNA interference (RNAi).

Increased production of ketone bodies, such as BOH and acetoacetate, has been shown to be associated with oxidative stress [27,28] and an important role of oxidative stress has been identified in the palmitate toxicity [18]. Therefore, genes with large (positive or negative) regression coefficients to ketone body release could suggest pathways and genes relevant to the cytotoxicity. The genes with the largest positive regression coefficients to B-OH are listed in Table 3. The complete list of regression coefficients can be found in the additional data file 2. Genes relevant to ROS production, fatty acid metabolism, and detoxification of lipid peroxidation products were found to have high regression coefficients. GSTM5 was most positively related to the BOH (and in turn, the cytotoxicity), while other isoforms of GST, e.g., GSTM4 and GSTM1 (listed in additional file 2), had negative regression coefficients. The basal level of GSTM5 in the liver is much lower than those of GSTM1 and GSTM4 [30]. Therefore, in spite of a large positive coefficient of GSTM5, the combined negative effect of GSTM1 and GSTM4 on the cytotoxicity is expected to dominate. Similarly, aldehyde dehydrogenase 1 (ALDH1A1) was found to have a positive relation to cytotoxicity, while ALDH1A3 (listed in additional file 2) had a negative regression coefficient. We found that the basal levels of ALDH1A3 were about 10^-5^-times lower than that of ALDH1A1 (Table 4). Therefore, the cytotoxic effects of ALDH1A1 would be greater than the cytoprotective effects of ALDH1A3. Indeed, over-expression of ALDH1A3 had no significant effect on the cytotoxicity or the caspase-3 activation by palmitate (not shown). Two NADH dehydrogenases had highly positive regression coefficients, indicating a positive role of these genes in the cytotoxicity. Their combined coefficient was greater than any other gene, indicating that these genes may play very important roles in the toxicity. NADH dehydrogenase complex, or complex I of the electron transport chain, is a main site of superoxide production [30,31]. In the course of electron transfer some of the activated oxygen is released as superoxides or H_2_O_2 _[31]. Their selection suggested involvement of ROS in the toxicity. Indeed, elevated ROS production was observed in cells treated with palmitate (see section 4 b ii).

Intracellular TG was identified to have significantly negative association to the toxicity. In agreement with this, the channeling of palmitate to TG has been shown to protect the cells from its toxic effects [29]. Thus, genes with high positive regression coefficients to intracellular TG accumulation may be potential targets for cytoprotection. Such genes are listed in Table 5. A complete list of regression coefficients is available in the additional data file 3. Endothelial membrane protein 3 (EMP3) was the gene with greatest positive coefficient, indicating that a reduction in the expression of this gene would be cytotoxic and vice-versa. The function of EMP3 is not clearly understood, it is known to be involved in cell growth, differentiation, and apoptosis [32,33]. Many genes involved in glutathione metabolism had positive regression coefficients. Examples of genes related to glutathione that were selected included glutamate-cysteine ligase, catalytic subunit (GCL-c), glutathione S-transferase M4 (GSTM4), microsomal glutathione S-transferase 1 (MGST1), glutathione S-transferase A2 (GSTA2) and glutathione reductase (GSR). The selection of these genes indicated a cytoprotective role of these genes, and corroborated with the role of oxidative stress in the toxicity. Finally, some genes involved in regulation of glycolysis such as hexokinase 1 (HK1) and glucokinase (hexokinase 4) regulatory protein (GCKR) had large positive regression coefficients, indicating that upregulation of glycolysis may be cytoprotective. Exposure to FFAs is associated with a reduction in cellular energetics, e.g., a decrease in glycolytic enzymes [34,35] and a reduction in ATP synthesis due to mitochondrial uncoupling [36]. This reduction in cellular ATP levels may play a role in the toxicity [37] or exacerbate it. Under these conditions, increasing glycolysis may provide an alternative route for ATP synthesis and reduce the toxicity.

### 4. Experimental validations

Hepatocytes are resistant to the cytotoxic effects of TNF-α due to rapid upregulation of cytoprotective genes, mediated in part by the activation of NF-κB by TNF-α. It is for this reason that most of the previous studies on the cell death caused by TNF-α employ a secondary insult, such as transcriptional inhibition or glutathione depletion [4,15]. The observation of the dependence of TNF-α toxicity on that of palmitate suggests that the toxicity of palmitate can act as a secondary/additional insult. Because the saturated free fatty acid (palmitate) was found to have the greatest toxicity of all the treatments and the toxicity of TNF-α depended on the effect of palmitate, the subsequent validations were conducted for the palmitate conditions.

#### a. Validation of GSEA results

##### a.i. The role of mitochondria

**Figure 9:**
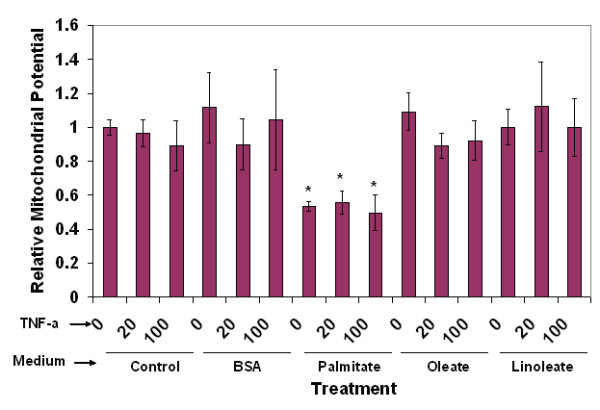
Effect of FFAs and TNF-α on the mitochondrial potential. HepG2 cells were exposed to different types of FFA and/or TNF-α for 24 h. After the treatments, the cells were exposed to 5 μM JC-1 dye in DMEM for 30 minutes, and then washed with PBS. Fluorescence of the cells were read using a microplate reader. Cells were excited at 488 nm, the green fluorescence was measured as emission at 527 nm, and the red fluorescence was measured as emission at 590 nm. The mitochondrial potentials were measured as the ratio of red to green fluorescence. Data presented as average +/- s.d. of n = 9 from three independent experiments. *, significantly lower than control.

Mitochondria are known to be central to cell death pathways, and most cytotoxic insults are associated with a loss of mitochondrial integrity. Mitochondrial membrane potential is an indicator of mitochondrial integrity and functioning. GSEA suggested involvement of these organelles in the cytotoxicity. We measured the mitochondrial potentials in response to the various treatments, as shown in Figure 9. Palmitate-treatment significantly decreased the mitochondrial potential. The loss of mitochondrial integrity will cause many deleterious effects, including the breakdown of energy generation, increased ROS production, and the release of apoptogenic factors such as cytochrome c from the mitochondria [38].

##### a.ii. Lack of involvement of *de novo *ceramide synthesis

*De novo *ceramide synthesis has been suggested to play an important role in the toxicity of saturated FFAs to beta cells and cardiomyocytes. However, the ceramide metabolism pathway was not found to be significantly enriched according to GSEA, suggesting that this pathway may not play an important role in the toxicity of the saturated FFAs to hepatoma cells. To test this prediction, we treated the cells with palmitate in the presence of an inhibitor of *de novo *ceramide synthesis, Fumonisin B1 (FB1). Treatment with this inhibitor reduced the ceramide levels significantly (by about 77%, Table 6). However, no change in the toxicity was observed. Similarly, treatments with higher concentrations of this inhibitor (up to 100 uM) had no effect on the toxicity of the saturated FFA. Therefore, these experiments corroborated the GSEA prediction that ceramide metabolism does not play an important role in the toxicity. These results were also in agreement with a previous study which found that hepatocyte cell death induced by the saturated FFAs is independent of ceramide synthesis [17,18].

#### b. Validating hierarchical model predictions

##### b.i. Important role of NADH dehydrogenase

**Figure 10:**
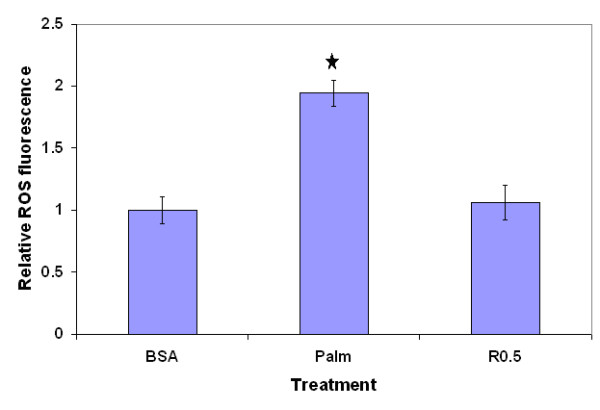
Complex I inhibitor decreases ROS level. HepG2 cells were pretreated with the inhibitor of mitochondrial complex I- rotenone (0.5 μM) for 30 minutes, and then exposed to 0.7 mM palmitate for 24 h. After the treatment, the cells were then washed with PBS, and exposed to 5 μM AM-DCFDA in DMEM for 30 min, following which the cells were washed again. The fluorescence was read by exciting the cells at 488 nm and reading the emission at 527 nm. Data presented as average +/- s.d. of n = 9 from three independent experiments. *, significantly higher than the control.

**Figure 11:**
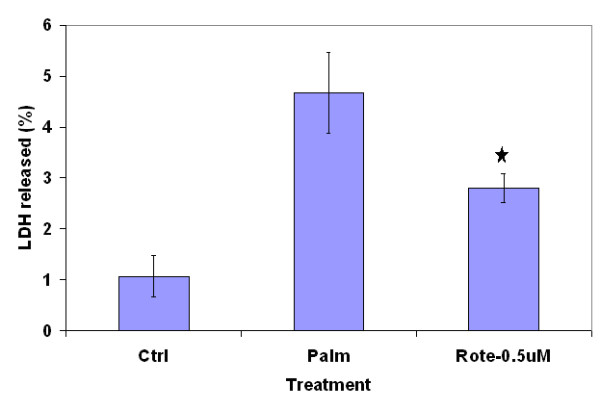
Complex I inhibitor decreases LDH release. HepG2 cells were pretreated with the inhibitor of mitochondrial complex I- rotenone (0.5 μM) for 30 minutes, and then exposed to 0.7 mM palmitate for 24 h. After the treatment, the media were collected and LDH released was measured. The cells were lyzed in 1% triton for 24 h to obtain the unreleased LDH. The LDH released was normalized to the total LDH (released + unreleased). Data presented as average +/- s.d. of n = 9 from three independent experiments. *, significantly lower than palmitate.

Exposure to palmitate has been shown to increase the ROS production in CHO cells [20]. We investigated the role of ROS production in the palmitate-toxicity in our case. As shown in Figure 10, the ROS levels were significantly increased in the palmitate-treated cells. In contrast, ROS levels were not increased in the cells treated with the unsaturated FFA, oleate, and treatments with radical scavengers significantly reduced palmitate toxicity [18]. This suggested that increased ROS production plays an important role in palmitate toxicity. We performed further studies with co-supplementation of FFAs. Exposure to 0.4 mM palmitate was also toxic to the cells (about 20% LDH release on day 2), while treatment with 0.4 mM palmitate and 0.3 mM oleate were non-toxic (LDH release < 1%). These results indicated that the production of ROS and LDH release were specific to palmitate treatment. NADH dehydrogenases (mitochondrial complex I) has been suggested as an important source of ROS. The hierarchical model identified that 2 isoforms of NADH dehydrogenases had important roles in the cytotoxicity of the FFA. To experimentally validate their role in the cytotoxicity, we measured the ROS level and LDH release in palmitate cultured cells pretreated with irreversible NADH dehydrogenase inhibitor. Pretreatment with the NADH dehydrogenase inhibitor rotenone (0.5 uM) significantly reduced the ROS levels (Figure 10) as well as the LDH release (Figure 11), confirming the role of NADH dehydrogenase in the observed toxicity as predicted by the model. The cytotoxicity was, however, not completely prevented by the complex I inhibitor, suggesting the involvement of other, ROS-independent, mechanisms in the cytotoxicity.

##### b.ii. The role of aldehyde dehydrogenases

**Figure 12:**
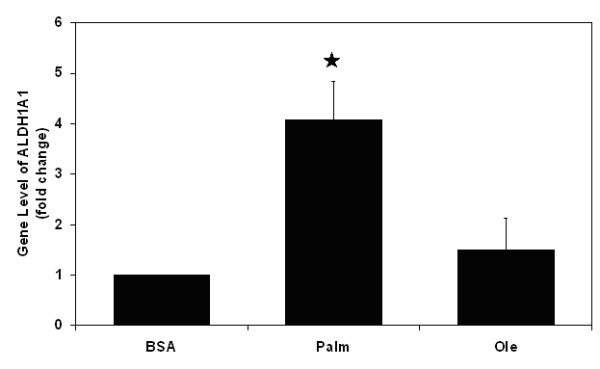
Effect of the FFAs on the levels of ALDH1A1. Cells were treated with 0.7 mM palmitate or oleate complexed to 4% BSA. Control cells were treated with just BSA. After 24 h of the treatment, RNA was isolated and the transcript of ALDH1A1 was quantified by RT-PCR. Data presented as mean +/- s.d. of n = 9 from 3 independent experiments.

**Figure 13:**
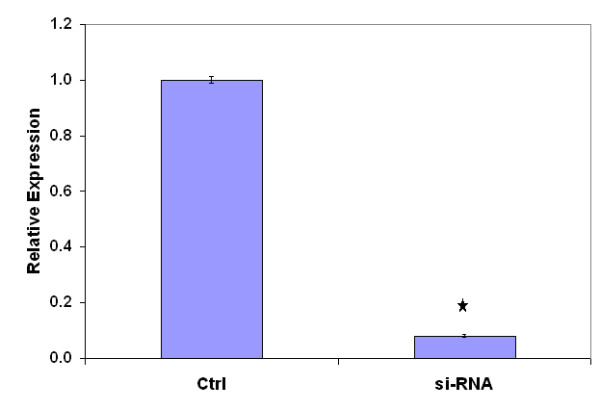
ALDH1A1 Silencing. Cells were transfected with siRNA targeting ALDH1A1, control cells were mock-transfected. The transfected cells were cultured in antibiotic-free medium for 24 h, followed by culture in medium containing 4% BSA for 24 h. Total RNA was extracted and the transcript levels of ALDH1A1 were measured by RT-PCR. Data presented as mean +/- s.d. of n = 9 from 3 independent experiments.

**Figure 14:**
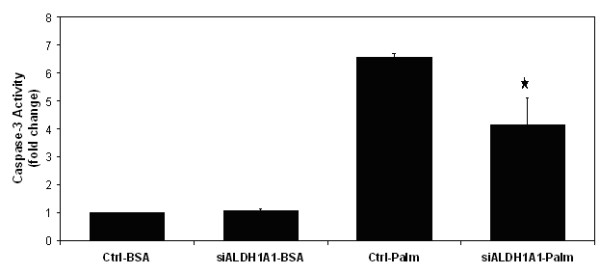
Effect of knock down of ALDH1A1 on the caspase-3 activity in response to palmitate. After culturing the cells in antibiotic-free medium for 24 h following transfection, cells were treated for 24 h with control (4% BSA) or palmitate (0.7 mM) media for another 24 h. The caspase-3 activities were measure with a fluorescence-based assay using a commercially available kit. Data presented as mean +/- s.d. of n = 9 from 3 independent experiments.

The analysis of regression coefficients suggested that many ALDH family members had positive association with the toxicity. Among these, ALDH1A1 was found to have the greatest positive regression coefficient to cytotoxicity (Table 3). Exposure to palmitate, but not to oleate, significantly increased the expression of ALDH1A1 (Figure 12). Therefore, we tested the effect of RNA interference (RNAi) of the ALDH1A1 gene on the toxicity. RNAi of ALDH1A1 significantly reduced its expression (Figure 13). The reduction in ALDH1A1 significantly reduced the caspase-3 activity of the cells in response to palmitate alone (Figure 14), confirming the cytotoxic role of ALDH1A1.

##### b.iii. The role of endothelial membrane protein 3 (EMP3)

**Figure 15:**
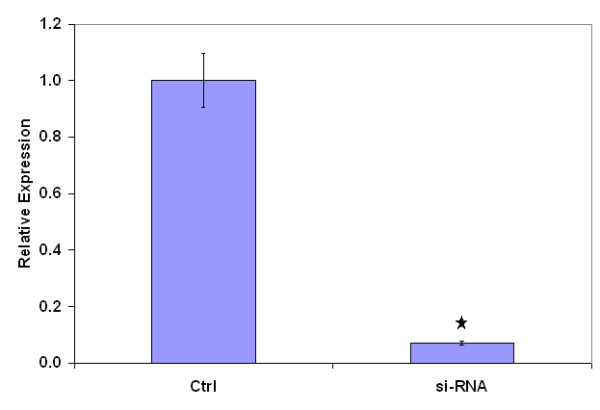
RNA interference of EMP3. Cells were transfected with siRNA targeting EMP3, control cells were mock-transfected. The transfected cells were cultured in antibiotic-free medium for 24 h, followed by culture in medium containing 4% BSA for 24 h. Total RNA was extracted and the transcript levels of EMP3 were measured by RT-PCR. Data presented as mean +/- s.d. of n = 9 from 3 independent experiments.

**Figure 16:**
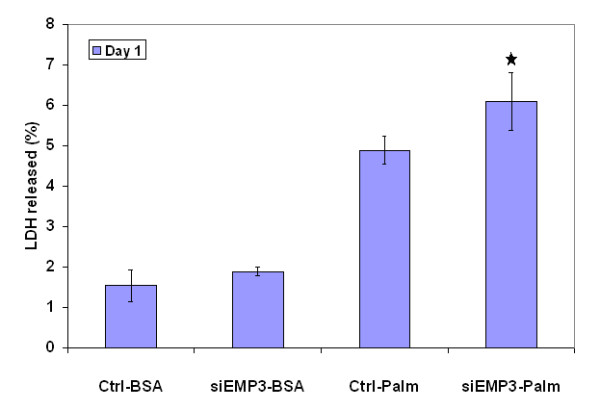
Effect of knock down of EMP3 on the LDH release in response to palmitate. After culturing the cells in antibiotic-free medium for 24 h following transfection, cells were treated for 24 h with control (4% BSA) or palmitate (0.7 mM) media for another 24 h and the LDH release was measured. Data presented as mean +/- s.d. of n = 9 from 3 independent experiments.

EMP3 was identified as a potentially cytoprotective gene. This suggested that knocking down EMP3 would increase the toxicity of palmitate. To test this prediction, EMP3 was knocked down using RNAi (Figure 15). The RNA interference of EMP3 significantly increased the toxicity of palmitate treatment (Figure 16), verifying the predicted cytoprotective role of EMP3.

## Discussion

In this paper, a framework was developed to hierarchically integrate the metabolic and gene expression profiles to identify the genes which play important roles in determining the phenotypic responses. Application of this framework to the FFA and TNF-α induced cytotoxicity in HepG2 cells yielded novel targets to regulate the toxicity. The genes and pathways relevant to the cytotoxicity were identified and experimentally validated. For example, GSEA identified that mitochondrial alterations, but not ceramide synthesis, were associated with the toxicity. An advantage of incorporating GSEA analysis into our hierarchical approach is that incorporating knowledge-based information enhances the signal to noise ratio and the robustness of the analysis, and permits the detection of genes with modest changes [24]. As illustrated by 1) the identification of ceramide metabolism not as an important player in the observed toxicity, which was experimentally validated, and 2) the novel targets identified by the hierarchical approach, suggest that the GSEA pathway analysis can compensate for the limited number of replicates to provide useful information. The former prediction is supported by the observation of no effect on the palmitate-induced toxicity upon inhibition of ceramide synthesis. The MBPLS prediction of the role of NADH dehydrogenase in regulating cytotoxicity was validated with complex I inhibitor studies, while those of ALDH1A1 and EMP3 were confirmed by RNAi studies.

Identification of genetic changes that control the phenotypic responses is an area of active research. This requires identification of genes that regulate the altered metabolic/physiological changes. Among the simpler models to identify such genes are the multivariate linear regression models [39]. We also conducted multivariate linear regression analysis to relate the genetic and metabolic profiles, and found that the linear regression identified a much smaller number of genes relevant to a metabolite production or release which were assigned regression coefficients different from zero. For example, only 25 genes out of the 272 genes had weights different from zero in the linear regression model to predict BOH (see additional data file 4 for the list of genes). None of the important genes identified by MBPLS such as NADH dehydrogenases, glutathione S-transferases, ALDHs or EMP3 were selected. Linear regression model is ill-conditioned when the number of variables (genes) exceeds the number of observations (conditions), resulting in most of the regression coefficients taking on values of zero. MBPLS is a multivariate approach capable of modeling a large number of variables using a relatively small set of observations. It circumvents typical problems associated with the highly correlated and collinear nature of experimental data by projecting the data onto a few independent latent factors. These latent factors simplify the complex and diverse relationships by capturing the variable interactions contained in the original data in a new set of fewer unobserved/latent variables.

In this study, in addition to the generation of important information such as the roles of NADH dehydrogenases and the lack of involvement of *de novo *ceramide in the toxicity, two novel modulators of the palmitate-toxicity (ALDH1A1 and EMP3) were identified and validated. A notable point is the differences in the response of ALDH1A1 and EMP3 silencing. While silencing ALDH1A1 reduced caspase-3 activity significantly, it did not affect LDH release. On the contrary, silencing EMP3 increased LDH release without any affecting the caspase-3 activity. These differences in the cellular responses are most likely due to the cellular location of the corresponding proteins (cytosolic for ALDH1A1 and cell membrane for EMP3). Exposure to elevated levels of FFA would lead to increased omega oxidation, in which ALDHs play an important role. Omega oxidation can be a source of ROS and toxicity [40]. Under these conditions, ALDH knock-down would reduce the omega oxidation and hence the cytotoxicity. In addition to its cytotoxic roles, ALDH1A1 may have potentially cytoprotective effects mediated by the detoxification of reactive lipid aldehydes [41]. EMP3 is a protein of the peripheral membrane protein 22 (PMP22) family [32]. The proteins of this family have been suggested to play important roles in cell proliferation and apoptosis [33]. However, there has not been any study on the role of EMP3 in fatty acid toxicity. Its effect on the LDH release suggests the possibility that this protein may regulate membrane integrity.

## Conclusion

In summary, this paper illustrated how phenotypic, metabolic and genetic profiles can be integrated hierarchically to identify phenotype relevant genes and pathways and novel targets. This approach identified the involvement of ROS generation, altered fatty acid and energy, but not ceramide metabolism in the cytotoxicity. In addition, novel targets such as ALDH1A1 and EMP3 were identified to modulate the toxicity of saturated FFA. Thus, the integration of metabolic and genetic information provides a more comprehensive picture of the perturbations as well as novel targets to regulate cellular responses.

## Methods

### Materials

HepG2/C3A cells and Fetal Bovine Serum were purchased from American Type Culture Collection (ATCC, Manassas, VA). Dulbecco's modified Eagle's medium with high glucose and no pyruvate (DMEM), Penicillin-Streptomycin (P/S), phosphate buffered saline (PBS, pH 7.4) and trizol reagent were purchased from Invitrogen (Carlsbad, CA). Fatty acid free bovine serum albumin (BSA) was purchased from MP Biomedicals (Chillicothe, OH). Sodium salts of all the fatty acids (palmitate, oleate and linoleate) were purchased from Sigma Aldrich chemical company (St. Louis, MO). 6-carboxy-2',7'-dichlorodihydrofluorescein diacetate, di(acetoxymethyl ester) (DCFDA dye) was obtained from Molecular Probes (Eugene, OR). Recombinant human TNF-α was from Peprotech (Rocky Hill, NJ).

### Cell culture

One million HepG2/C3A cells were seeded into each well of a 6-well culture plate. The cells were cultured in 2 ml of medium containing DMEM supplemented with 10% fetal bovine serum (FBS) and 2% Penicillin-streptomycin (P/S). Cells were incubated at 37°C and in 10% CO_2 _atmosphere. After the cells reached confluence, the medium was replaced with 2 ml of the chosen medium, either HepG2, or the FFA medium containing 0.7 mM palmitate, oleate or linoleate; or the FFA-TNF-α medium. The fatty acids chosen are the most prevalent in the class of saturated (palmitate), monounsaturated (oleate) and polyunsaturated (linoleate) fatty acids in the plasma. The concentration of fatty acids chosen (0.7 mM) is commonly found in conditions of obesity. The FFAs were dissolved in 4% fatty acid-free BSA. Therefore, in addition to the HepG2 medium control, 4% fatty acid-free BSA in HepG2 medium was used as another control. TNF-α was added from a 100ug/ml stock in deionized water to make the desired final concentrations of either 20 or 100 ng/ml.

### Cytotoxicity measurement

The cytotoxicity of the treatments was measured as the fraction of lactate dehydrogenase (LDH) released into the medium. LDH is a cytosolic enzyme which leaks out into the medium on exposure to toxic chemicals. Thus, the fraction of LDH released into the medium provides an accurate estimate of the cytotoxicity. For the LDH measurements, the cells were cultured in different media for 24 h and the supernatant was collected. Cells were washed with phosphate buffered saline (PBS) and kept in 1% triton-X-100 in PBS for another 24 h at 37°C. Cell lysate was then collected, vortexed for 15 seconds and centrifuged at 7000 rpm for 5 minutes. Cytotoxicity detection kit (Roche Applied Science, Indianapolis, IN) was used to measure the LDH release. LDH released was normalized to the total LDH (released + lyzed). The LDH released into the medium was normalized to total LDH, as shown in the equation below

%LDHrelease=LDH(medium)LDH(total)×100
 MathType@MTEF@5@5@+=feaafiart1ev1aaatCvAUfKttLearuWrP9MDH5MBPbIqV92AaeXatLxBI9gBaebbnrfifHhDYfgasaacH8akY=wiFfYdH8Gipec8Eeeu0xXdbba9frFj0=OqFfea0dXdd9vqai=hGuQ8kuc9pgc9s8qqaq=dirpe0xb9q8qiLsFr0=vr0=vr0dc8meaabaqaciaacaGaaeqabaqabeGadaaakeaacqGGLaqjcqWGmbatcqWGebarcqWGibascqWGYbGCcqWGLbqzcqWGSbaBcqWGLbqzcqWGHbqycqWGZbWCcqWGLbqzcqGH9aqpdaWcaaqaaiabdYeamjabdseaejabdIeaijabcIcaOiabd2gaTjabdwgaLjabdsgaKjabdMgaPjabdwha1jabd2gaTjabcMcaPaqaaiabdYeamjabdseaejabdIeaijabcIcaOiabdsha0jabd+gaVjabdsha0jabdggaHjabdYgaSjabcMcaPaaacqGHxdaTcqaIXaqmcqaIWaamcqaIWaamaaa@596A@

### Caspase-3 assay and TUNEL staining

Cells were treated with different FFA in the presence and absence of TNF-α. Caspase-3 activity was measured using a commercially available fluorescence-based assay kit from Biomol, according to manufacturer's protocol. Data were normalized to protein measurements from parallel experiments. For TUNEL staining, cells were cultured in Labtek Chamber Slide System. TUNEL staining was performed using the Dead-End fluorimetric TUNEL system from Promega Biosciences, according to manufacturer's protocol and imaged under a fluorescence microscope (Leica).

### Measurement of metabolic uptake and production

The net uptake or production of a species was calculated by the difference in the concentration of the specie in the medium, before and after the treatment. The concentrations of metabolites were measured using enzymatic assays or HPLC. The amino acids were measured by HPLC using the AccQTag method (Waters) according to the manufacturer's protocol. Briefly, the media collected after treatments were de-proteinized by adding 4X acetonitrile (by volume) and keeping on ice for 1 h, after which the samples were centrifuged and the supernatant was derivatized using the reagent in the AccQTag kit as per the instructions given. The samples were analyzed with HPLC on a Waters 2690 separations module, using an AccQTag column (Waters) and fluorescence detection (Waters detector). Intracellular triglycerides were measured by lysing the cells with 1% triton-X-100 for 24 h and measuring the triglycerides by an enzymatic assay kit from Sigma. Glucose, lactate and glycerol were measured using enzymatic analysis kits from Sigma. Free fatty acid half micro kit (Roche Diagnostics) was used to find the concentration of free fatty acids in the medium. Beta-hydroxybutyrate (BOH) released was measured using a kit from Stanbio. Acetoacetate was measured using enzymatic assay [42].

### Fisher's discriminant analysis

FDA identifies the projection axis that maximizes the ratio of the between-group and the within-group variations. Details on the FDA algorithm can be found in [23]. FDA was applied to identify which among the 27 measured metabolic uptake/production contributed to the separation of the different phenotypes (cytotoxic versus nontoxic). Because there were 2 classes (toxic and non-toxic), a single discriminating vector is sufficient to separate them [43]. The relative importance of the metabolites was identified by the projection of the metabolites in the new discriminant vector space.

### Microarray analyses

Cells were cultured in 10 cm tissue culture plates until confluence and then exposed to different treatments for 24 h. RNA was isolated with the Trizol reagent. The gene expression profiles were obtained with the cDNA microarrays. The microarray analyses were conducted at the Van Andel Institute, Grand Rapids, MI. The protocols are available online at [44]. There were two biological replicates for each condition and each replicate was measured with the Cy3 and Cy5 dyes (i.e. there were two technical replicates for each biological replicate). The microarray data has been deposited at the GEO website [45], with a query number of GSE5441.

### Measurement of reactive oxygen species

Confluent cells were treated with the control medium or 0.7 mM palmitate for different time periods. Positive control cells were treated with 2 mM H_2_O_2 _for 1 h at 37°C. Measurement of ROS was performed by flow cytometry, using 6-carboxy-2',7'-dichlorodihydrofluorescein diacetate, di(acetoxymethyl ester) (C-2938, Molecular Probes, Eugene, OR) dye. A 5 mM stock solution of the dye was prepared in DMSO, and diluted to the 5 umol/L in DMEM. After the desired treatment times, the cells were exposed to the dye for 30 min, following which the cells were washed and imaged under a fluorescence microscope. Quantitative measurements were made by reading the fluorescence of the cells in a microplate fluorimeter with excitation at 488 nm and emission at 527 nm.

### Measurement of ceramide

The cells were treated with 0.7 mM palmitate in the presence or absence of 20 μM Fumonisin B1 (FB1) for 24 h, following which the lipids were extracted by the method of Bligh and Dyer [46]. The C-16 ceramide in the samples were then quantified with LC-MS as per a previously published protocol [47].

### Gene Set Enrichment Analysis (GSEA) of the gene data

GSEA integrates *a priori *knowledge of a gene's functional role with the expression data to detect concerted expression changes in a set of genes responsible for producing a phenotype. The software GSEA-P, from [48], was used for the GSEA analysis. Thirty seven gene sets were selected from the molecular signature database, MsigDB [25] functional gene group c2, as shown in Table 1. These sets included 10 metabolic pathways, 26 signal pathways and 1 cellular component. An enrichment score of a gene set S characterizes whether the set of genes randomly distributed across the list or falls mainly at the bottom or top of the list. The null hypothesis that a gene set S randomly distributes across the ranked gene list was tested with Kolmogorov-Smirnov test, with the statistical significance value estimated through 1000 random permutations of the phenotype label. The gene sets with a high significance of enrichment are considered important in separating the distinct phenotypes.

### Integrating the gene expression and metabolic profiles

MBPLS is a hierarchical multivariate analysis method [49,50], where the variables are divided into different blocks based upon *a priori *knowledge, for example according to different stages of a process [49] or different metabolic pathways in a cell [26]. For details of the MBPLS algorithm, refer [26]. In the MBPLS model, the different gene sets identified by GSEA formed the blocks of the MBPLS. This ensured that the blocks were separated according to the functional role of the genes. Block scores were extracted from each block to predict the metabolic uptake/productions. This facilitated the identification of an important block (gene set) to a metabolic flux, and identified the important genes within the block. Important genes sets were identified by evaluating the weights of each block and importance of individual genes were then further identified by evaluating the regression coefficients of the genes within the block. Two latent variables were selected to be extracted from each block based upon the prediction accuracy of the MBPLS model. The N-way toolbox [51] was applied to conduct the MBPLS modeling.

### RNA interference and reverse transfection

Silencer^® ^Validated siRNAs targeting human EMP3 and ALDH1A1 mRNA were purchased from Ambion (Austin, TX). The synthesized oligonucleotides for siRNA of EMP3 are 5'-GUCCCUGAAUCUCUGGUACtt-3' and 5'-GUACCAGAGAUUCAGGGACtc-3', and the synthesized oligonucleotides for siRNA of ALDH1A1 are 5'-GGAACAGUGUGGGUGAAUUtt-3' and 5'-AAUUCACCCACACUGUUCCtg-3'. Reverse transfection of siRNA was performed. In general, siRNA and the transfection reagent, Lipofectamine RNAiMAX (Invitrogen, Carlsbad, CA), were diluted in Opti-MEM (Invitrogen) devoid of any serum and antibiotics and added to the 6-well plates. After 20 minutes, the same numbers of HepG2 cells in antibiotic free media were plated in each well and incubated at 37°C. After 24 h of transfection, the HepG2 cells were cultured for another 24 hours in regular media with other additives, for example palmitate. Then cells were harvested, washed twice with phosphate-buffered saline and lysed.

### Overexpression and forward transfection

The ALDH1A3 plasmid, pCMV6-XL4-hALDH1A3, was purchased from Origene (Rockville, MD). Transient transfection was performed according to the Lipofectamine 2000 (Invitrogen) method. In general, the HepG2 cells were seeded in 6-well plates and cultured until reaching 80% confluency. Before transfection, the cells were washed twice with phosphate buffered saline, and medium was replaced with 2 ml of Opti-MEM medium. 1 μg/well of pCMV6-XL4-hALDH1A3 was then mixed with 5ul/well of Lipofectamine 2000 in Opti-MEM, and 20 minutes later, the mixture was added to the wells. After 6–12 hours of transfection, the cells were then cultured in regular media, treated with other additives like palmitate or TNF-α, and harvested after the treatment.

### Real-time quantitative RT-PCR analysis

Total RNA was extracted from cells with an RNeasy mini kit and depleted of contaminating DNA with RNase-free DNase (Qiagen, Valencia, CA). Equal amounts of total RNA (1 μg) were reverse-transcribed using an iScript cDNA synthesis kit (Bio-RAD). The first-strand cDNA was used as a template. The primers used for quantitative RT-PCR analyses of human EMP3 (5'-GTGGTCTCAGCCCTTCACA-3' and 5'-ACGTGCAGTCGTACCAGAGA-3'), human ALDH1A1 (5'-AGCCTTCACAGGATCAACAGA-3' and 5'-GTCGGCATCAGCTAACACAA-3'), human ALDH1A3 (5'-GCCCTTTATCTCGGCTCTCT-3' and 5'-CGGTGAAGGCGATCTTGT-3') and human GAPDH (5'-AACTTTGGTATCGTGGAAGGA-3' and 5'-CAGTAGAGGCAGGGATGATGT-3') were synthesized by Operon Biotechnologies, Inc. (Huntsville, AL). RT-PCR was performed in 25-μl reactions using 1/10 of the cDNA produced by reverse transcription, 0.2 μM each primer, 1 X SYBR green supermix from Bio-RAD, and an annealing temperature of 57°C for 40 cycles. Each sample was assayed in three independent RT reactions and triplicate reactions each and normalized to GAPDH expression. Negative controls included the absence of enzyme in the RT reaction and absence of template during PCR. The cycle threshold (*C*_*T*_) values corresponding to the PCR cycle number at which fluorescence emission in real time reaches a threshold above the base-line emission were determined using MyIQ™ Real-Time PCR Detection System.

## Abbreviations

**ACAC **acetoacetate

**ALDH **aldehyde dehydrogenase

**BOH **beta-hydroxy butyrate

**EMP3 **endothelial membrane protein 3

**FFA **free fatty acid

**GSEA **gene set enrichment analysis

**GST **glutathione-S transferase

**HNF **hepatic nuclear factors

**LDH **lactate dehydrogenase

**MBPLS **multi-block partial least squares analysis

**NASH **non-alcoholic steatohepatitis

**NF-κB **nuclear factor kappa B

**PPAR **peroxisome proliferator activated receptors

**ROS **Reactive oxygen species

**SREBP **sterol receptor element binding protein

**TNF-α **tumor necrosis factor alpha

## Authors' contributions

ZL conceived the methodology and performed part of the experiments and wrote the manuscript. SS performed the cell culture, LDH measurement, microarray measurement, caspase, TUNNEL and metabolite measurement, wrote part of the manuscript. SM performed cell culture and extracted RNA for microarray measurement. XY performed ALDH and EMP3 experiment and wrote part of the manuscript. PN, JR, BH prepared, did QC/QC and performed cDNA microarry measurements. CC conceived the study and supervised the experiment and writing of the manuscript. All authors read and approved the final manuscript.

## Supplementary Material

Additional data file 1 Metabolic profiles. The data is a table of 27 measured metabolic uptake/productions. Click here for file

Additional data file 2 BOH regression coefficients. Additional data file 2 is a table listing the regression coefficients of genes to BOH. Click here for file

Additional data file 3 TG regression coefficients. Additional data file 3 is a table listing the regression coefficients of genes to TG. Click here for file

Additional data file 4 BOH linear regression coefficients. Additional data file 4 is a table listing the regression coefficients to BOH with the linear regression model. Click here for file

## Figures and Tables

**Table 1:** Gene sets used in the GSEA analysis

**Metabolic pathways**	
Glycolysis	Fatty acid metabolism
TCA	Oxidative phosphorylation
PPP pathway	Beta-oxidation
Fructose	Sphingoglycolipid
Fatty acid biosynthesis	Glutathione

**Signal pathways**	

Bcl2 family_and_reg_network	Map_kinase
Caspase	Mapkk
Cell death	Ppar-alpha
CeramidePathway	Ppar-gamma
CrebPathway	S1P signaling
NF-kB	Programmed cell death
p38	ERK1 Erk2 Mapk pathway
Akt	JNK
EGF	TNFalpha
EIF2	Stress Pathway
EIF4	TNFR1 pathway
Electron Transport Chain	TNFR2 pathways
ERK pathway	ROS

**Cellular Component**	

Mitochondria	

**Table 2:** Enriched gene sets identified by GSEA analysis

**Gene Sets**	**SIZE**	**NES**	**NOM p-val**
Electron Transport Chain	43	-1.998	0.000
Fatty acid metabolism	33	-1.824	0.000
Glycolysis	55	-1.765	0.000
Oxidative Phosphorylation	30	-1.890	0.004
ROS	26	-1.741	0.009
PPP pathway	15	-1.644	0.010
Cell death	15	-1.631	0.010
ERK pathway	31	-1.620	0.016
Fatty acids beta-oxidation	7	-1.580	0.020
Fatty acid biosynthesis	5	-1.574	0.021
TCA	15	-1.631	0.023
Fructose	19	-1.599	0.026
ERK1 Erk2 Mapk pathway	29	-1.542	0.034
Glutathione	23	-1.520	0.036
Mitochondria	229	-1.945	0.002

Size: number of genes in the gene set; NES: normalized enrichment score; NOM p-val: nominal P value.

**Table 3:** Top ranked genes relevant to beta-hydroxbutyrate

**Accession #**	**Regression coefficient**	**Gene Name**
N56898	3.89E-02	glutathione S-transferase M5 (GSTM5)
AA664101	3.64E-02	aldehyde dehydrogenase 1 family, member A1 (ALDH1A1)
AA406536	3.33E-02	NADH dehydrogenase (ubiquinone) Fe-S protein 1, 75kDa (NADH-coenzyme Q reductase) (NDUFS1)
AA680322	3.15E-02	NADH dehydrogenase (ubiquinone) 1 alpha subcomplex, 4, 9kDa (NDUFA4)
AA443630	2.93E-02	aldehyde dehydrogenase 3 family, member B2 (ALDH3B2)
T72259	2.72E-02	cytochrome P450, subfamily IIA (phenobarbital-inducible), polypeptide 7 (CYP2A7), transcript variant 2
H59758	2.31E-02	v-raf murine sarcoma 3611 viral oncogene homolog 1 (ARAF1)
AA035384	2.21E-02	succinate dehydrogenase complex, subunit D, integral membrane protein (SDHD)
AA055585	2.20E-02	core promoter element binding protein (COPEB)
T52484	2.20E-02	Nerve growth factor, beta polypeptide (NGFB)

Top 10 genes with regression coefficients of highest absolute values. The regression coefficients of all genes range from -0.042 to 0.039.

**Table 4:** Basal Levels of ALDH1A1 and ALDH1A3 in control cells (relative to GAPDH) measured by RT-PCR

	**Average**	**SD**
**GAPDH**	1	0.02
**ALDH1A1**	4.91E-02	5.92E-03
**ALDH1A3**	1.92E-07	7.34E-08

**Table 5:** Top ranked genes positively related to TG

**Accession #**	**Regr. Coeff.**	**Gene Name**
W73810	2.23E-02	epithelial membrane protein 3 (EMP3), mRNA.
AA485272	1.95E-02	hexokinase 1 (HK1), transcript variant 5, nuclear gene encoding mitochondrial protein, mRNA.
N45129	1.82E-02	glutamate-cysteine ligase, catalytic subunit
T67006	1.80E-02	glucokinase (hexokinase 4) regulatory protein (GCKR), mRNA.
AA486570	1.79E-02	glutathione S-transferase M4 (GSTM4), transcript variant 3, mRNA.
R66006	1.72E-02	acyl-Coenzyme A dehydrogenase, long chain (ACADL), mRNA.
AA495936	1.58E-02	microsomal glutathione S-transferase 1 (MGST1), transcript variant 1a, mRNA.
H93482	1.50E-02	glutamate-cysteine ligase, catalytic subunit
T73468	1.45E-02	glutathione S-transferase A2 (GSTA2), mRNA.
AA676663	1.34E-02	acyl-Coenzyme A dehydrogenase, C-2 to C-3 short chain (ACADS), nuclear gene encoding mitochondrial protein, mRNA.

The regression coefficients of all genes range from -0.027 to 0.023

**Table 6:** Effect of ceramide inhibition on the toxicity of palmitate

Treatment	C16 Ceramide (pmol/mg protein)	LDH released (%)
Control (BSA)	0.63 ± 0.28 *	1.27 ± 0.48 *
Palmitate	24.44 ± 6.39	4.71 ± 1.67
Fumonisin B1	6.80 ± 2.85 *	5.15 ± 1.38

*, significantly lower than palmitate
